# MR evaluation of tetralogy of Fallot patients after surgical repair: relationship between aortic dilation and aortic regurgitation

**DOI:** 10.1186/1532-429X-14-S1-P112

**Published:** 2012-02-01

**Authors:** Alexander W Keedy, David Naeger, Michael D Hope, Karen Ordovas

**Affiliations:** 1 Radiology, UCSF, San Francisco, CA, USA

## Background

To evaluate the prevalence of aortic morphologic and hemodynamic abnormalities and their relationship with aortic regurgitation in surgically corrected of Tetralogy of Fallot (TOF) patients.

## Methods

We retrospectively identified 97 consecutive patients with surgically corrected TOF and 26 controls without TOF or aortic or cardiac abnormalities who underwent cardiac MR evaluation. The ascending aortic diameter was measured at four levels and diameter ratios were calculated for morphologic characterization. Aortic pulse wave velocity (PWV) and aortic regurgitation were calculated from velocity-encoded cine data. Aortic size was compared between the TOF and control groups, as well as to population based data. The relationship between regurgitation and aortic imaging parameters was assessed.

## Results

Compared to population-based data, 62% of TOF patients had a dilated sinus of Valsalva, compared to none of the controls (p<0.001). TOF patients had larger aortic diameters at all measured levels compared to the controls, most prominent at the sinus of Valsalva where the median diameter was 2.20 versus 1.55 cm/m2, respectively (p<0.001). The median sinus of Valsalva/proximal arch diameter ratio was 1.45 in TOF patients, versus 1.26 in the control group (p<0.001). Aortic PWV was higher in patients with TOF compared to controls (median=4.43 vs 3.8m/s; p=0.03). Between TOF patients with (>15%) and without (<15%) significant aortic regurgitation, there was no statistically significant difference in aortic size, shape and PWV (p<0.05 for all comparisons). In a multivariate regression model, aortic regurgitation was associated with age (p=0.003), but not aortic diameter (p=0.61) nor PWV (p=0.39).

## Conclusions

Ascending aortic dilation is common in TOF patients and PWV is increased in TOF patients compared to controls. While common, these measures correlate poorly with cardiac function and further study is needed to define which imaging parameters are clinically relevant in surgically corrected TOF patients.

## Funding

None.

